# Extracellular matrix composition affects outgrowth of dendrites and dendritic spines on cortical neurons

**DOI:** 10.3389/fncel.2023.1177663

**Published:** 2023-06-14

**Authors:** Archana Sharma, Katherine E. Hill, Jean E. Schwarzbauer

**Affiliations:** Department of Molecular Biology, Princeton University, Princeton, NJ, United States

**Keywords:** decellularized ECM, fibronectin, tenascin-C, cortical neurons, dendrites, spines

## Abstract

The composition of the extracellular matrix (ECM) in nervous tissue plays an important role in controlling neuronal outgrowth and synapse development. Changes in both protein and glycosaminoglycan components of the ECM occur with tissue injury and may affect neuron growth. To investigate neuron responses to alterations in fibronectin (FN), a major component of the wound ECM, we grew cortical neurons on cell-derived decellularized matrices composed of wild type FN (FN^+/+^) or of a mutant form of FN (FN^Δ/+^) from which the III_13_ heparin-binding site had been deleted by CRISPR-Cas 9 gene editing. The most significant effect of the mutant FN was a reduction in dendrite outgrowth. Not only were dendrites shorter on mutant FN^Δ/+^-collagen (COL) matrix than on wild type (FN^+/+^-COL) matrix, but the number of dendrites and dendritic spines per neuron and the spine densities were also dramatically reduced on FN^Δ/+^-COL matrices. Mass spectrometry and immunostaining identified a reduction in tenascin-C (TN-C) levels in the mutant matrix. TN-C is an ECM protein that binds to the III_13_ site of FN and modulates cell-matrix interactions and has been linked to dendrite development. We propose that TN-C binding to FN in the wound matrix supports dendrite and spine development during repair of damaged neural tissue. Overall, these results show that changes in ECM composition can dramatically affect elaboration of neurites and support the idea that the ECM microenvironment controls neuron morphology and connectivity.

## Introduction

The neuronal microenvironment changes dramatically at sites of tissue injury and repair. In the central nervous system (CNS), tissue injury disrupts the organization of neurons and their connections to other cells while also causing degradation of the supportive extracellular matrix (ECM) (Shechter and Schwartz, 2013). Non-neural cell types enter the injury and resident astroglia become activated to promote reparative processes. The wound ECM consists of blood proteins, mainly fibrin(ogen) and plasma fibronectin (FN), which are supplemented over time with tissue-produced FN and collagens (Midwood et al., 2004b). These ECM proteins are assembled into a fibrillar network that supports cell adhesion and migration in areas where the natural ECM has been lost. Their accumulation changes the stiffness of the microenvironment which may affect the functions of neurons and astroglia. Certain wound ECM proteins found in glial scars have inhibitory action in CNS healing (Kjell and Gotz, 2020). Specifically, chondroitin sulfate proteoglycans (CSPG) appear to block new nerve cell growth (Jones et al., 2003) and proteins known to modulate cell-ECM interactions such as tenascin-C (TN-C) are up-regulated (Roll and Faissner, 2019).

Clearly, there are significant changes in the ECM at CNS injury sites, but much remains to be learned about how these changes promote or inhibit repair and recovery of function. One important question relates to the extension of new axons and dendrites, the neuronal processes that are involved in neuronal connectivity and formation of synaptic connections with other neurons (Kurshan et al., 2014). Along dendrites, actin-filled dendritic spines protrude and are involved in the development of synaptic contacts and synaptic plasticity (Dent et al., 2011; Miermans et al., 2017). Spines undergo morphological changes in response to neuronal activity (Hotulainen and Hoogenraad, 2010). Spine stability and remodeling is important for normal function and spine disruption may contribute to CNS diseases (Levy et al., 2014; Runge et al., 2020). Axons, dendrites, and spines normally develop through productive interactions with their ECM (Barros et al., 2011; Long and Huttner, 2019). How these processes develop within the changed ECM of a wound is not well understood.

Cultured fibroblasts assemble a FN and type I collagen-rich fibrillar matrix that resembles the matrix formed during wound repair (Gardiner, 2011; Wehner et al., 2017; Sharma and Schwarzbauer, 2022). Decellularized ECM derived from fibroblasts provides a suitable microenvironment for determining how the ECM contributes to neuronal outgrowth (Singh et al., 2014; Harris et al., 2017; Sharma and Schwarzbauer, 2022). FN matrix is foundational in that FN fibrils provide the template for incorporation of many other proteins into the ECM including collagens, proteoglycans, and growth factors (Singh et al., 2010; Maeda, 2015). The glycosaminoglycan (GAG) chains of CSPGs and HSPGs bind to the HepII heparin-binding domain of FN suggesting that this domain may participate in interactions that can inhibit neuron outgrowth. TN-C also binds to this domain and some of its modulatory activities are known to depend on binding to FN (Midwood and Schwarzbauer, 2002; Midwood et al., 2004a). Perhaps reducing these interactions might affect neuron-ECM interactions, either by changing the strength of cell attachment or by altering the extension of axons or dendrites. For this reason, we developed a decellularized matrix from cells in which the III_13_ heparin-binding module in HepII was deleted by CRISPR-Cas 9 gene editing. This module is the primary site of GAG binding to FN and also interacts directly with TN-C and several neurotrophic factors (Barkalow and Schwarzbauer, 1994; Chiquet-Ehrismann, 2004; De Laporte et al., 2013; Martino et al., 2014).

Here we examine outgrowth of neurites from cortical neurons on FN-collagen (FN-COL) decellularized matrices derived from wild type (FN^+/+^) and III_13_-deleted (FN^Δ/+^) fibroblasts. We describe differences in axons, dendrites and dendritic spines from cortical neurons on these two matrices and identify changes in ECM composition with deletion of the heparin-binding site. Our results suggest that the HepII domain of FN is important for development of a microenvironment conducive to neuron functionality and nerve repair.

## Materials and methods

### Dissection of cortices and cell culture

All the animal experiments were conducted according to the guidelines of Princeton University Institutional Animal Care and Use Committee (IACUC). Sprague-Dawley rats (Hilltop Labs Incorporated) were euthanized, E16.5-E17.5 embryos were removed, and cortices were harvested from the brains (Pacifici and Peruzzi, 2012; Schildge et al., 2013; Sahu et al., 2019). After dissection, cortices could be stored up to one week in Hibernate-E reagent (Gibco). Cortical neurons and glial cells were dissociated from cortex using 0.25% trypsin (Gibco) and cultured on decellularized matrices in neuronal growth media which consists of Neurobasal medium, 1% B-27 medium supplement, 200 mM glutamine, 1% penicillin/streptomycin (all purchased from Gibco) and 100 ng/ml Nerve Growth Factor (NGF) (Gibco). In all experiments, 5,000 cells dissociated from a cortex were seeded per well of a 24-well dish. NIH 3T3 cells (ATCC) were cultured in Dulbecco’s modified Eagle’s medium (DMEM) (Hyclone) supplemented with 10% bovine calf serum (BCS) (Hyclone) and 1% antibiotic/antimycotic (Corning). All the other chemicals used in the study were of analytical grade.

### Decellularized wild type and mutant extracellular matrices

Extracellular matrices were prepared from NIH 3T3 cells using a modified decellularization procedure (Sharma and Schwarzbauer, 2022). Wild type NIH 3T3 fibroblasts (FN^+/+^) and by heterozygous fibroblasts expressing a fibronectin (FN) mutant missing the III_13_ heparin-binding site (FN^Δ/+^), which was deleted using CRISPR-Cas 9 gene editing (Hill, 2022; Lovett et al., 2023), were grown with 50 μg/ml ascorbate (Sigma) and used to produce wild type FN^+/+^-COL and mutant FN^Δ/+^-COL decellularized matrices. For immunoblots and mass spectrometry, wild type and mutant decellularized matrices were solubilized with lysis buffer (50 mM TEAB pH 7.4 + 5% SDS). Protein concentrations were determined by BCA assay and equal amounts of total protein from all samples were electrophoresed on a 6% polyacrylamide/SDS gel. The presence of FN and collagen I were confirmed by immunoblotting by using R184 rabbit anti-FN polyclonal antiserum (1:50,000) against type III_1–6_ (Saunders and Schwarzbauer, 2019; Sharma and Schwarzbauer, 2022). Type I collagen was detected with anti-collagen type I antibody A1 (PA2140-2, Boster Biological Technology). TN-C was detected with R759 rabbit anti-TN-C antiserum (1:100, R759 was generated in-house) (Vega et al., 2020) followed by goat anti-rabbit IgG (1:600, Invitrogen). Mass spectrometry analysis of these matrices has been performed as described (Sharma and Schwarzbauer, 2022).

Deletion of the exons encoding the III13 domain was confirmed by PCR of genomic DNA PureLink Genomic DNA mini Kit) and RT-PCR of RNA extracted from wild type and mutant fibroblasts (Vega et al., 2020) and using primers homologous to sequences flanking III13 (Lovett et al., 2023).

### Immunofluorescence and microscopy

FN^+/+^-COL and FN^Δ/+^-COL decellularized matrices were fixed in 3.7% formaldehyde in PBS and then stained with DAPI (1:500, Sigma), rabbit anti-FN antiserum (R184, diluted 1:100 or rabbit anti–collagen I/COLI A1 polyclonal antibody (diluted 1:500, Boster Biological Technology) followed by Alexa fluor 488 and Alexa fluor 568 goat anti-rabbit IgG (1:600, Invitrogen), respectively. Cortical neurons were seeded on decellularized FN^+/+^-COL and FN^Δ/+^-COL matrices for the indicated times and then fixed as above and permeabilized with 1% Triton X-100 in PBS at RT for 15 min (Sharma and Schwarzbauer, 2022). Neurons and microtubules were visualized using monoclonal NeuN (1:1000, MA5-33103, Invitrogen) and monoclonal anti-α-tubulin antibody (1:500, Clone-B-5-1-2, Sigma) followed by Alexa fluor 488 goat anti-mouse IgG (1:600, Invitrogen). Actin filaments were stained with Texas-red phalloidin (1:100, Invitrogen). Focal adhesions were visualized by anti-kindlin-2 monoclonal antibody (clone 3A3, 1:100, Millipore) followed by Alexa fluor 488 goat anti-mouse IgG (1:600, Invitrogen). Samples on coverslips were mounted using ProLong Gold Antifade reagent (Invitrogen). A Nikon Eclipse Ti inverted microscope with Hamamatsu digital camera was used to capture images. Mean fluorescence quantification was performed using ImageJ. Background fluorescence was removed from randomly selected images using a rolling ball radius of 50 (Vega et al., 2020). Images were analyzed using Nikon NIS Elements and ImageJ software.

### Analyses of axons, dendrites, and spines

Fluorescence images of cortical neurons on decellularized wild type FN^+/+^-COL and mutant FN^Δ/+^-COL matrices were captured at 24 and 72 h. We did not measure beyond this time because, at 72 h of culture, both the number of overlapping neurites and the possibility of changes made to the matrix by interacting cells are limited. Quantifications of axon length, dendrite length, dendrite width, numbers of dendrites per neuron, and numbers of spines per neuron were performed using neurons stained with monoclonal anti-α-tubulin and Texas red-phalloidin. Percentage of NeuN-positive cells with and without axons were analyzed. Neurites that were clearly separated with both ends visible were counted. Axon and dendrite lengths were measured by tracing actin-positive membrane projections with the freehand line tool in ImageJ (Sharma and Schwarzbauer, 2022). For neurons with multiple neurites, the longest neurite is the putative axon and is referred to as an axon throughout this study. Overlapping or intertwined neurites were excluded from measurements.

Actin filled projections extending from neuronal soma are considered as dendrites and projections on dendrites are considered as spines. Analysis of dendrite outgrowth, the number of dendrites/neuron, dendritic spines, the spine count/neuron on wild type and mutant decellularized matrices were measured using “dendritic spine counter” by ImageJ. Dendrites and spines were also analyzed at higher magnification (60×). Multiple images of immunostained samples were taken at random at each time point. Cortical neurons on decellularized matrices were analyzed from two independent experiments.

### Statistical analyses

All the graphs were made with GraphPad Prism 8 software. Statistical comparisons between samples at different time points were performed using either an unpaired *t*-test or one-way ANOVA followed by Bonferroni post-test, as indicated in the figure legends. The results were considered statistically significant when *P* ≤ 0.05. All data are presented as mean ± standard error of the mean (SEM).

## Results

### Characterization of wild type and mutant decellularized matrices

**FIGURE 1:**
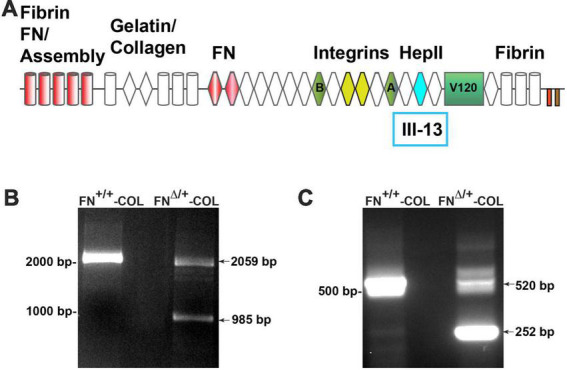
**(A)** Schematic diagram of a fibronectin (FN) subunit [adapted from Schwarzbauer and DeSimone (2011)]. FN is composed of three types of repeats: type I (cylinders), type II (diamonds), and type III (hexagon). Major domains are indicated for FN assembly (type I_1–5_); binding to collagen, FN (III_1–2_), and integrins (III_9–10_); the C-terminal cysteine pair for intermolecular dimer formation; variable domains (A,B, V120); and the HepII domain (III_12–14_). The site for heparin binding is within type III_13_ highlighted in aqua and was deleted in one allele of FN. PCR analysis of genomic DNA **(B)** and cDNA **(C)** from wild type and mutant cells was performed using primers from sites that flank III_13_ in the FN sequence. MW markers are indicated to the left of each gel image.

**FIGURE 2:**
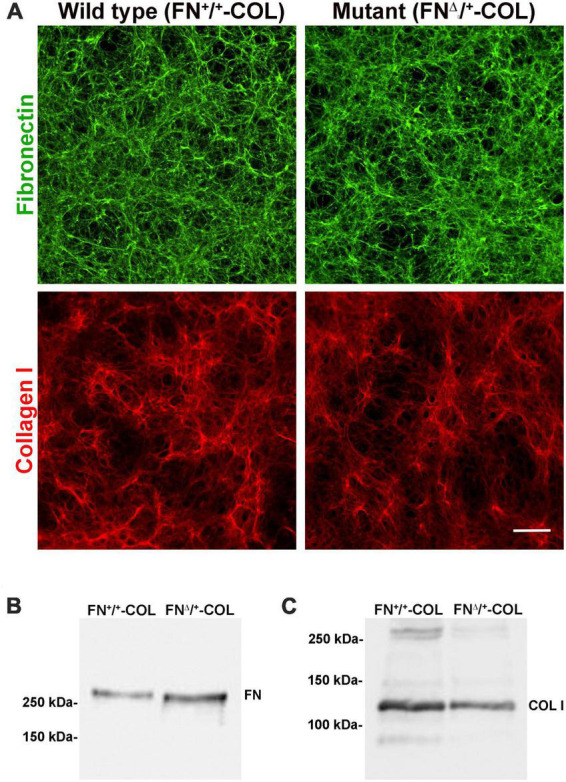
Three-dimensional decellularized fibrillar wild type (FN^+/+^-COL) and mutant (FN^Δ/+^-COL) matrices visualized by immunofluorescence. **(A)** Decellularized matrices were stained with R184 rabbit anti-FN antiserum (green) and with polyclonal anti-COL I antibody (red) followed by goat anti-rabbit IgG. Scale bar = 100 μm. **(B,C)** Decellularized matrices were solubilized in SDS buffer and protein concentrations were determined using a BCA assay. Equal amounts of total protein were separated on 6% polyacrylamide/SDS gels and analyzed by immunoblotting with anti-FN (R184) antiserum **(B)** and anti-COL I antibody **(C)**.

To determine the role of FN interactions with glycosaminoglycans in neurite outgrowth, we developed a mouse fibroblast cell line in which FN lacks the III_13_ module containing the primary heparin-binding site in FN’s Hep II domain (Figure 1A). The exons encoding this module were deleted using CRISPR-Cas 9 gene editing and cells in which one allele had been edited were cloned to generate a heterozygous III_13_ deletion (FN^Δ/+^) (Hill, 2022; Lovett et al., 2023). PCR analysis of genomic DNA isolated from wild type (FN^+/+^) and mutant (FN^Δ/+^) cells shows that the mutant cells are heterozygous for deletion of III_13_. Using primers in sequences flanking the deletion site, the wild type product at 2059 bp was detected in both wild type and mutant DNAs while a second band at 985 bp representing deletion of the III_13_ exons was detected in mutant DNA (Figure 1B). To confirm the expression of FN lacking III_13_, RT-PCR was performed on RNA isolated from wild type (FN^+/+^) and mutant (FN^Δ/+^) cells and shows the presence of two bands at 520 and 252 bp in the edited cells compared to a single wild type band at 520 bp in the parental cells (Figure 1C). Cell-derived decellularized ECMs were prepared from cells grown in the presence of ascorbate to promote type I collagen production (Sharma and Schwarzbauer, 2022). Immunofluorescence staining of decellularized matrices illustrates similar organizations and amounts of fibrils in FN^+/+^ and FN^Δ/+^ cells with both anti-FN and anti-collagen antibodies (Figure 2A). Similar levels of FN and collagen I were also detected in immunoblots of solubilized decellularized matrix samples (Figures 2B, C and Supplementary Table 3; Sharma and Schwarzbauer, 2022). Because FN^Δ/+^ cells produce two forms of FN, the dimeric FN secreted by those cells is a mixture of wild type and mutant dimers, while all FN subunits from wild type cells contain the III_13_ module (Schwarzbauer et al., 1989). FN and type I collagen are the major ECM proteins in the matrices, hence the name FN-COL.

### Axon outgrowth on FN^+/+^-COL and FN^Δ/+^-COL ECMs

**FIGURE 3:**
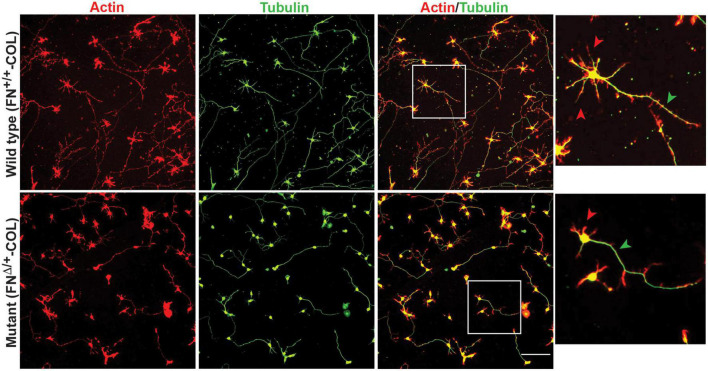
Representative images of cortical neurons cultured on decellularized wild type (FN^+/+^-COL) **(top)** and mutant (FN^Δ/+^-COL) **(bottom)** matrices at 72 h. Neurons were stained as indicated with phalloidin for actin filaments and anti-tubulin monoclonal antibody for microtubules. Boxed regions in the actin/tubulin merged images were enlarged and are shown to the right. Red and green arrowheads indicate dendrites and axons, respectively, extending from the neurons. Scale bar = 100 μm.

**FIGURE 4:**
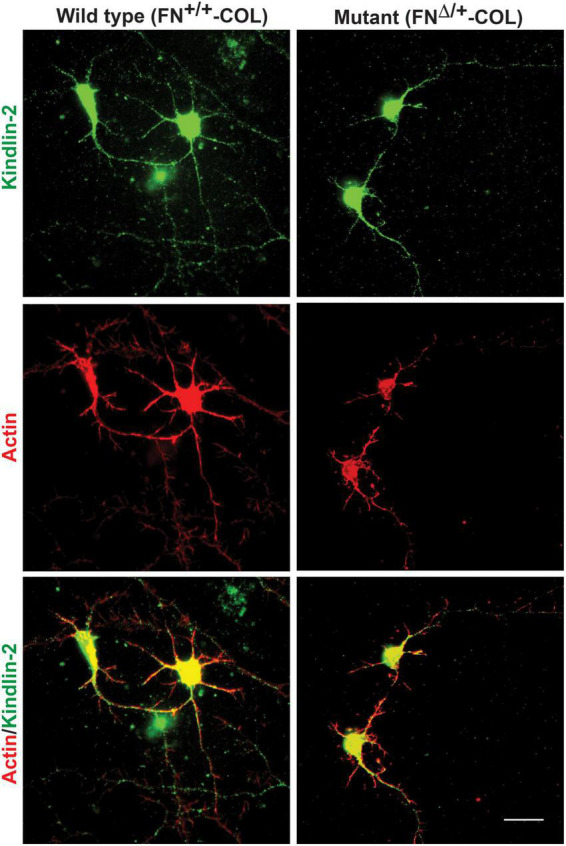
Distribution of kindlin-2 and actin filaments within dendrites and axons. Cortical neurons on wild type or mutant matrices were stained with anti-kindlin-2 antibody followed by fluorescein-goat anti-mouse IgG and co-stained with Texas-red phalloidin. Merged images are shown at the bottom. Scale bar = 20 μm.

**FIGURE 5:**
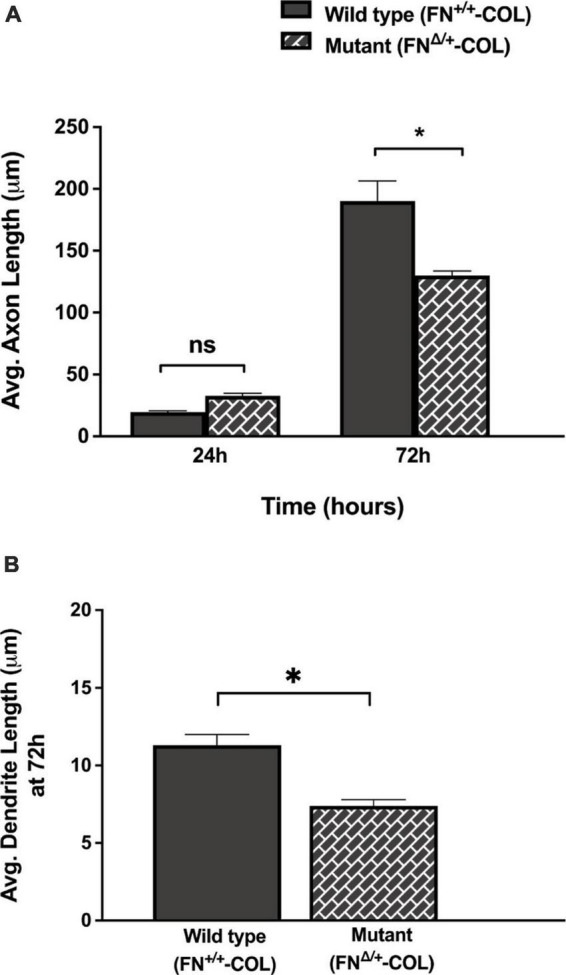
Lengths of axons and dendrites on decellularized matrices as indicated were measured with the help of Image J software by using a freehand line tool. **(A)** 44 and 63 axons were measured on FN^+/+^-COL and FN^Δ/+^-COL matrices, respectively, at 24 and 72 h; **(B)** 205 and 172 dendrites were measured on FN^+/+^-COL and FN^Δ/+^-COL matrices, respectively, at 72 h. Average lengths were calculated for each condition and are expressed as mean ± SEM. Statistical comparisons of data from two independent experiments were performed using **(A)** one-way ANOVA (*P* ≤ 0.001) followed by Bonferroni post-test (*P ≤ 0.05; ns = not significant) or **(B)** unpaired *t*-test (*P ≤ 0.05). Additional information on axons and dendrites is presented in Supplementary Table 1.

Embryonic rat cortical neurons were seeded on the decellularized ECMs and allowed to attach and extend neurites over 72 h. Each neuron extended a single long axon and multiple shorter dendrites which were easily visualized by co-staining microtubules and actin filaments (Figure 3). Neurons in the isolated cell population were identified by expression of the neuronal marker NeuN (Gusel’nikova and Korzhevskiy, 2015; Supplementary Figure 1A). About 85% of the cells in the cortical cell isolate were NeuN positive demonstrating they are neurons (Supplementary Figures 1B, C). Of the NeuN-positive cells, 96 and 88% extended axons on wild type (FN^+/+^-COL) and mutant (FN^Δ/+^-COL) matrices, respectively. To further characterize neuron adhesion to matrices, we analyzed localization of the integrin-associated protein kindlin-2, which is apparently the only kindlin expressed in neurons (Montanez et al., 2008; Tan et al., 2012). Kindlin coordinates with talin to activate integrins and promote cell adhesion (Theodosiou et al., 2016; Nieuwenhuis et al., 2018). Staining neurons with anti-kindlin-2 antibodies showed a punctate distribution along actin-filled neurites on both matrices (Figure 4). Together, the NeuN and kindlin-2 data show that neurons interact productively with both of these matrices. Axon lengths were measured by tracing from the cell body to the axon tip (Figure 5A). At 24 h, axons were very short with no significant difference between axons on wild type and mutant matrices. Axons were much longer by 72 h and showed a significant difference between matrices with longer axons detected on wild type matrix (Supplementary Table 1).

### Dendrite outgrowth is affected by ECM composition

**FIGURE 6:**
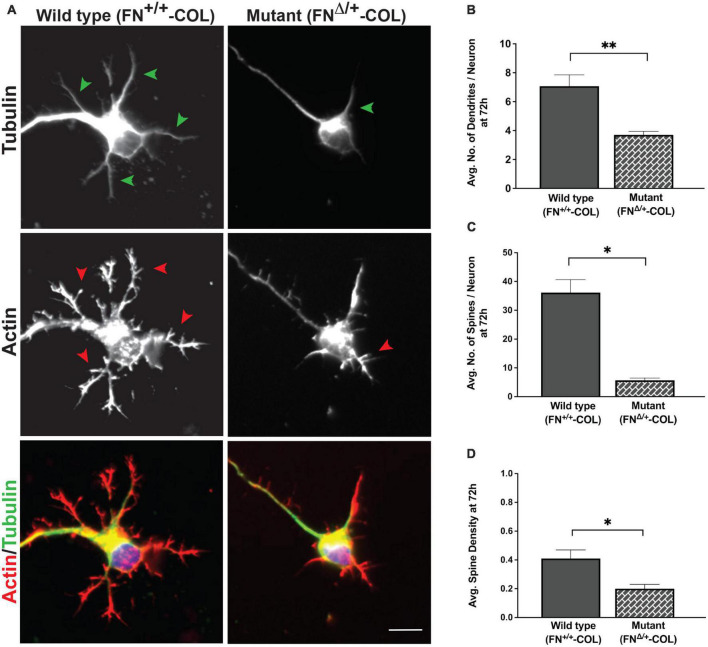
Dendrite outgrowth from cortical neurons on decellularized wild type (FN^+/+^-COL) and mutant (FN^Δ/+^-COL) matrices. **(A)** Representative images are shown of neurons with dendrites (green arrowheads) and dendritic spines (red arrowheads) stained for tubulin and actin at 72 h. Merged green and red images are shown at the bottom. Scale bar = 10 μm. **(B)** Dendrites (numbers in Figure 5B) and **(C)** spines (985 and 259 on FN^+/+^-COL and FN^Δ/+^-COL matrices, respectively) were counted and the calculated numbers per neuron are compared. **(D)** Spine densities (spines per dendrite length) were calculated and graphed. **(B–D)** Statistical comparisons between samples were performed using unpaired *t*-test of measurements from two independent experiments. Values are mean ± SEM; **P ≤ 0.01, *P ≤ 0.05. Total numbers (n) of dendrites and spines measured on each matrix are listed in Supplementary Table 2.

A more dramatic difference was detected between these matrices when dendrite outgrowth was quantified. First, one can clearly see that many more dendrites were extended from neuron soma on FN^+/+^-COL matrix compared to FN^Δ/+^-COL matrix (Figure 3 and Supplementary Figure 2). Second, average dendrite length was about 35% shorter on mutant matrix (Figure 5B and Supplementary Table 1) but dendritic width was not different on these matrices (Supplementary Table 2). By counting the number of dendrites, we determined that neurons on a wild type matrix produced almost twice as many dendrites as cells on mutant matrices (Figures 6A, B and Supplementary Table 2). These data show that decellularized FN^+/+^-COL matrices are more effective at supporting dendrite extension compared to FN^Δ/+^-COL matrices. Co-staining with phalloidin and anti-FN antibodies indicates overlap between dendrites and FN fibrils (Supplementary Figure 3) and suggests that interactions with the matrix may contribute to differences in dendrite numbers.

Dendritic spines are essential protrusions that indicate sites for synapses and form functional connections with axons of other neurons (Pchitskaya and Bezprozvanny, 2020; Runge et al., 2020). We evaluated the effects of FN^+/+^-COL and FN^Δ/+^-COL matrices on the formation of dendritic spines using dendritic spine counter software (Supplementary Figure 4). Examples of typical dendrites with spines are shown in Figure 6A. Number of spines/neuron was measured on both decellularized matrices. On FN^Δ/+^-COL matrix, the number of spines per neuron was about 6-fold less than on FN^+/+^-COL matrix after 72 h (Figure 6C and Supplementary Table 2). This matrix-dependent difference is not due to fewer or shorter dendrites since the spine density (spines per micron of dendrite) is also lower on the FN^Δ/+^-COL matrix compared to FN^+/+^-COL matrix (Figure 6D and Supplementary Table 2). These results show that changes in ECM composition related to reducing the number of heparin binding sites in FN dramatically affect dendrite and dendritic spine outgrowth.

### Differences in protein composition of decellularized matrices

**FIGURE 7:**
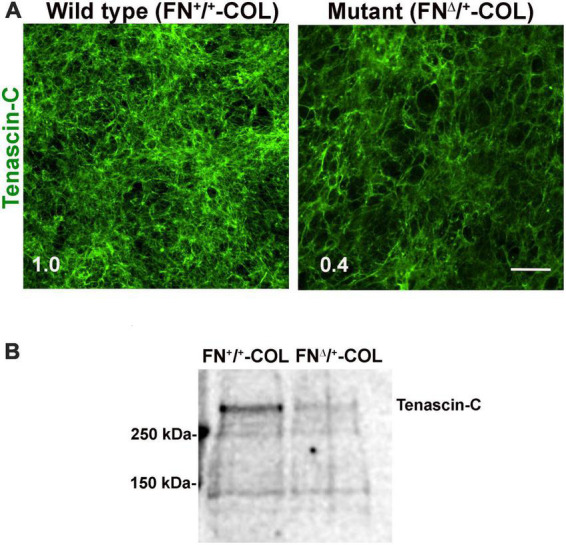
**(A)** Decellularized wild type (FN^+/+^-COL) and mutant (FN^Δ/+^-COL) matrices were stained with R759 rabbit anti-TN-C antiserum followed by goat anti-rabbit IgG. Mean fluorescence intensities were calculated from eight randomly selected fields per condition. Relative difference between TN-C in wild type and mutant matrices is indicated in the bottom left corner. Scale bar = 100 μm. **(B)** Matrices were solubilized and analyzed as in Figure 2B using R759 antiserum for the immunoblots. MW markers are indicated on the left.

To determine if there are differences in protein composition of the wild type (FN^+/+^-COL) and mutant (FN^Δ/+^-COL) matrices that might contribute to the morphological effects, mass spectrometry was performed on solubilized decellularized matrices. Spectral counts for ECM proteins are listed in Supplementary Table 3. FN is the most abundant protein in FN^+/+^-COL (Mao and Schwarzbauer, 2005) and FN^Δ/+^-COL matrices followed by HSPG2/perlecan. Types I, V, and VI collagens were detected and did not vary significantly between matrices. Two proteins that did change were type XII collagen, which increased in the mutant matrix, and TN-C, which decreased. TN-C is known to bind to the HepII domain of FN (Huang et al., 2001) and reportedly is involved in the dynamics of spines on neurons and in synaptic plasticity (Levy et al., 2014). Immunofluorescence staining of decellularized matrices with anti-TN-C antibodies showed a higher mean fluorescence in the FN^+/+^-COL matrix (Figure 7A). Less TN-C in the mutant matrix was confirmed by immunoblotting of solubilized decellularized matrices (Figure 7B). Thus, the mass spectrometry analyses identify changes in the composition of the ECM that we can link to the III_13_ module and that might contribute to the observed differences in neurite outgrowth and neuronal function.

## Discussion

In this study, we compared decellularized matrices produced by wild type NIH 3T3 fibroblasts and by heterozygous fibroblasts expressing a FN mutant missing the III_13_ heparin-binding site (FN^Δ/+^), which was deleted using CRISPR-Cas 9 gene editing. Decellularized matrices thus have either normal FN fibrils or fibrils containing a mixture of wild type (FN^+/+^) and mutant (FN^Δ/+^) FNs. We investigated the ability of these matrices (FN^+/+^-COL and FN^Δ/+^-COL) to promote axon and dendrite outgrowth by cortical neurons. The major difference between these matrices was in the extension of dendrites and dendritic spines. Twice as many dendrites and four times as many dendritic spines per neuron were extended on the FN^+/+^-COL matrix compared to FN^Δ/+^-COL matrix. These findings support the contention that the number of heparin binding sites on FN fibrils has a regulatory role in dendrite regeneration. Analysis of the protein composition of these matrices identified a reduction in the level of TN-C in the FN^Δ/+^-COL matrix. TN-C is a known FN binding protein, and our results indicating there is less TN-C in the mutant matrix suggest that dendrite formation may be affected by the level of TN-C incorporation into the matrix.

The fibrillar nature of decellularized FN-COL matrix resembles the organization of the ECM at sites of injury. Axon outgrowth has been shown to follow FN matrix fibrils during spinal cord repair (Wehner et al., 2017) and the presence of type I collagen in the matrix has been linked to changes in axonal protrusions and growth cone morphology of peripheral neurons (Sharma and Schwarzbauer, 2022). Here we identified a role for a specific FN domain in dendritic outgrowth of cortical neurons. Clearly, the wound ECM has an important role in controlling neurite outgrowth during nerve repair. Localization of integrin associated protein kindlin-2 on axons and dendrites and the overlap of actin staining with FN fibrils suggests that integrin adhesions mediate interactions with FN and/or COL fibrils in decellularized ECM. Our previous work also showed associations between neurites and FN matrix (Singh et al., 2014; Harris et al., 2017). Together these findings indicate that FN fibrils play an important role in directing neurite outgrowth in neural wound healing.

We found that the mutant FN^Δ/+^-COL matrix was less effective at promoting dendrite outgrowth and extension of dendritic spines. Extension of dendrites from neuronal cell body is required to make synaptic connections with axon termini of other neurons. In addition, dendritic spines are the sites for synapse formation and undergo morphological changes in response to neuronal activity. Dendrite and dendritic spine growth depend on actin cytoskeletal remodeling (Hotulainen and Hoogenraad, 2010) and are regulated by signals from neighboring cells and surrounding ECM molecules (Levy et al., 2014; Long and Huttner, 2019; Yang et al., 2023). At injury sites, neurons lose synaptic connections, and the native ECM is damaged or degraded. Newly deposited wound ECM proteins accumulate and can hinder neurite outgrowth. Our results indicate that a wild type FN^+/+^-COL matrix promotes dendrite and dendritic spine growth but that this growth is reduced if FN has fewer heparin-binding domains. Thus, the composition of FN in the matrix has a role in neuronal repair mechanisms.

Comparison of the composition of FN^+/+^-COL and FN^Δ/+^-COL matrices identified a reduction in TN-C levels in the mutant matrix. TN-C is an important glycoprotein for tissue repair and regeneration and modulates cell proliferation, motility, and ECM assembly by binding to the heparin-binding domain of FN and regulating intracellular signaling pathways (Huang et al., 2001; Midwood et al., 2004b; Tucic et al., 2021). TN-C is present in the ECM during neural development and in the adult CNS and has been linked to dendritic spine development and dendrite morphology (Dansie and Ethell, 2011; Levy et al., 2014). Our results suggest that TN-C may be affecting dendrites through its interaction with FN in the matrix. TN-C may be enhancing spine development and hence synapse formation. Fewer TN-C binding sites in the mutant FN matrix could then change the levels of TN-C and impact dendrite development. Another modulatory ECM protein, thrombospondin-1, can influence synapse formation and also binds to FN (Liu et al., 2001; Christopherson et al., 2005; Dansie and Ethell, 2011) suggesting a common mechanism for regulating wound ECM functions in nerve repair.

## Data availability statement

The original contributions presented in this study are included in this article/Supplementary material, further inquiries can be directed to the corresponding author.

## Ethics statement

The animal study was reviewed and approved by the Princeton University Institutional Animal Care and Use Committee (IACUC).

## Author contributions

AS, KH, and JS contributed to the conceptualization and methodology. AS performed the experiments. AS and JS contributed to the formal analysis, project administration, validation, and writing — original draft preparation. JS contributed to the funding acquisition, resources, and supervision. All authors contributed to the article and approved the submitted version.
