# Supplementary material for: Extracellular matrix composition affects outgrowth of dendrites and dendritic spines on cortical neurons
Source: Front Cell Neurosci. 2023 Jun 14;17:1177663. doi: 10.3389/fncel.2023.1177663 (PMC10300442; doi:10.3389/fncel.2023.1177663)
Supplement: Supplementary file 1 [file Data_Sheet_1.PDF]

Supplementary Materials

Extracellular matrix composition affects outgrowth of dendrites and dendritic spines on cortical neurons

Archana Sharma[1], Katherine E. Hill[1], and Jean E. Schwarzbauer[1*]

[1] Department of Molecular Biology, Princeton University, Princeton, NJ 08544, USA

***Correspondence:**
Jean Schwarzbauer, Ph.D.
Department of Molecular Biology
Princeton University
Princeton NJ 08544-1014
Ph: 609-258-2893
jschwarz@princeton.edu

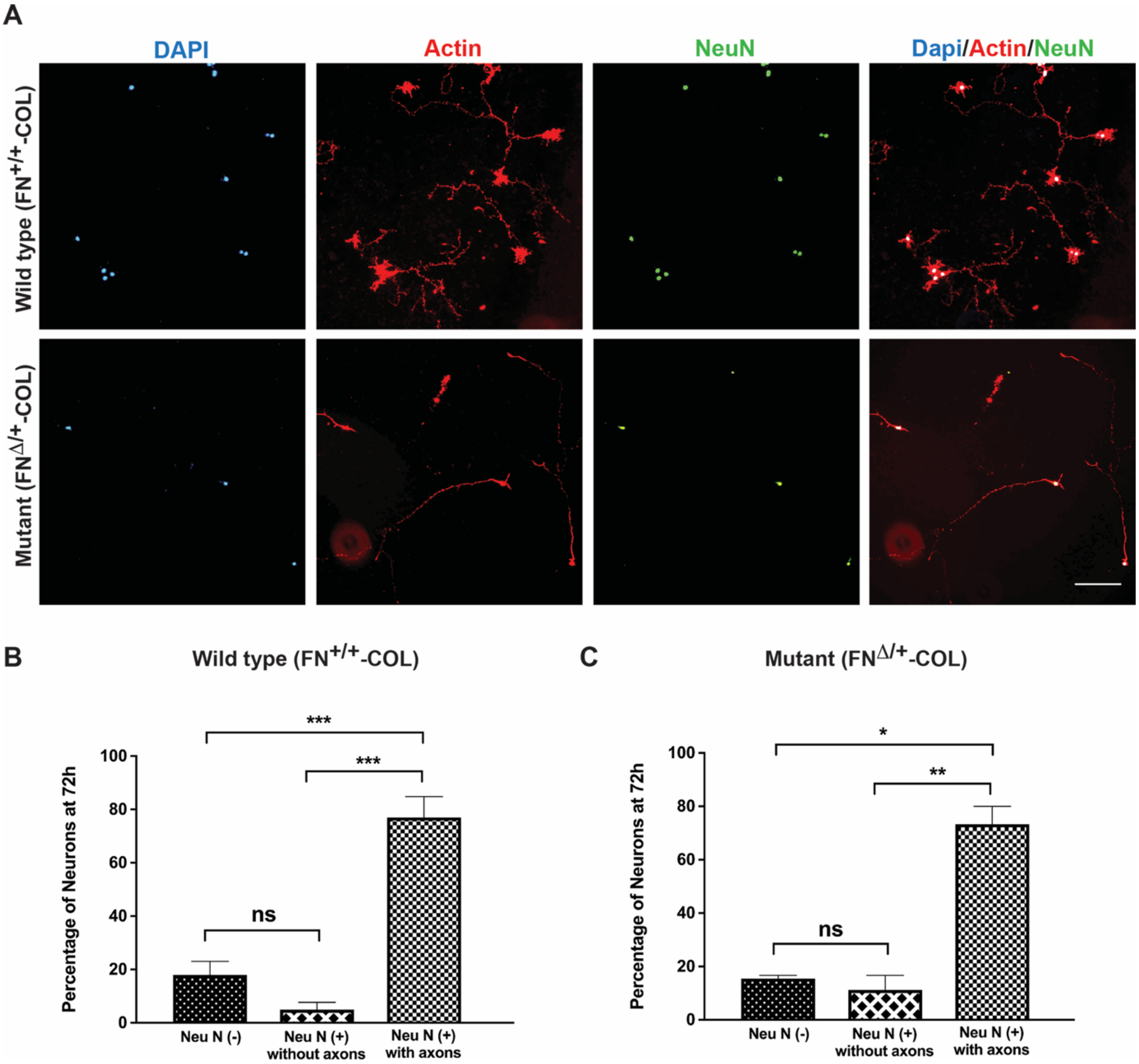

Supplementary Figure 1: Representative images of cortical neurons cultured on wild type ($FN^{+/+}$-COL) and mutant ($FN^{\Delta/+}$-COL) matrices after 72 h. (A) Cortical neurons were stained with Texas red-phalloidin for actin filaments, neurons with anti-NeuN monoclonal antibody and DAPI for nuclei. (B) and (C) percentage of Neu N-stained cells on both wild type ($FN^{+/+}$-COL) and mutant ($FN^{\Delta/+}$-COL) matrices. Numbers of neurons (n) measured on wild type and mutant matrices were 59 and 53, respectively, in two independent experiments. There was no statistical difference between the proportion of axon-positive neurons on wild type and mutant matrices. Statistical comparisons between samples were performed using one-way ANOVA followed by Bonferroni post-test. Values are mean ± SEM. *** $P \leq 0.001$; ** $P \leq 0.01$; * $P \leq 0.05$; ns= not significant ($P > 0.05$).

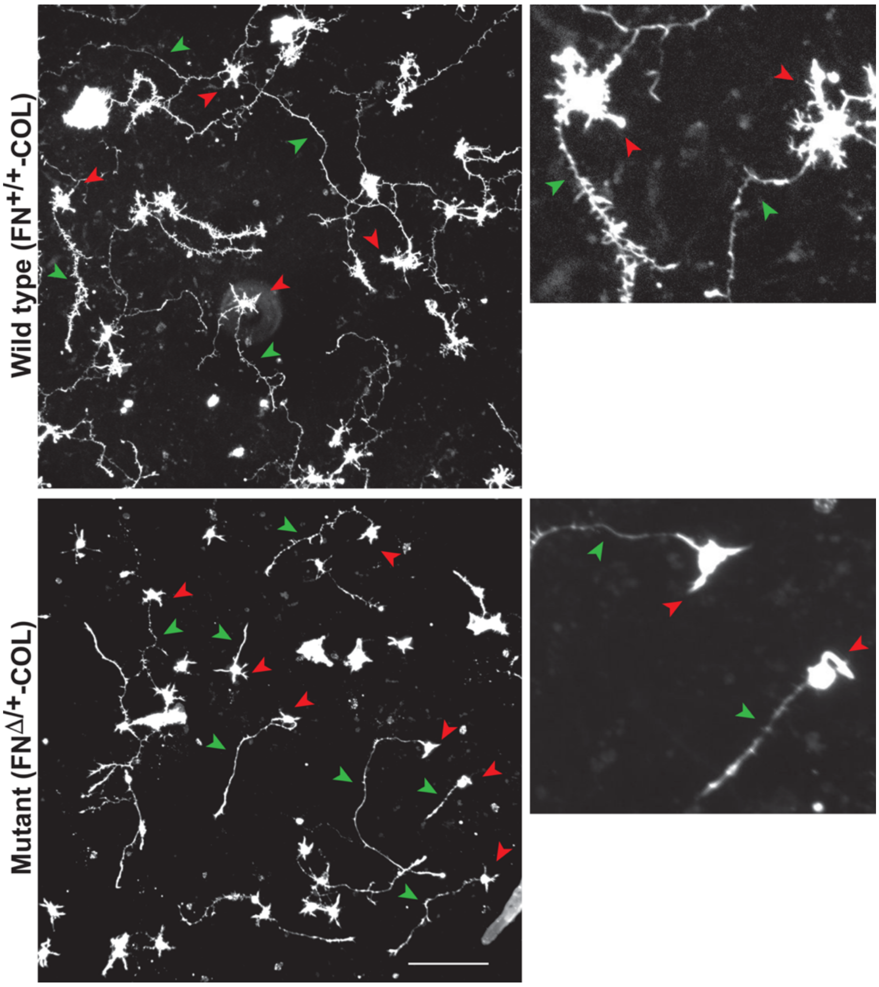

Supplementary Figure 2: Representative images of cortical neurons cultured on decellularized on wild type ($FN^{+/+}$-COL) and mutant ($FN^{\Delta/+}$-COL) matrices at 72 h. Cortical neurons were stained with Texas red-phalloidin for actin filaments. Green and red arrowheads indicate axons and dendrites, respectively, extending from the neurons.

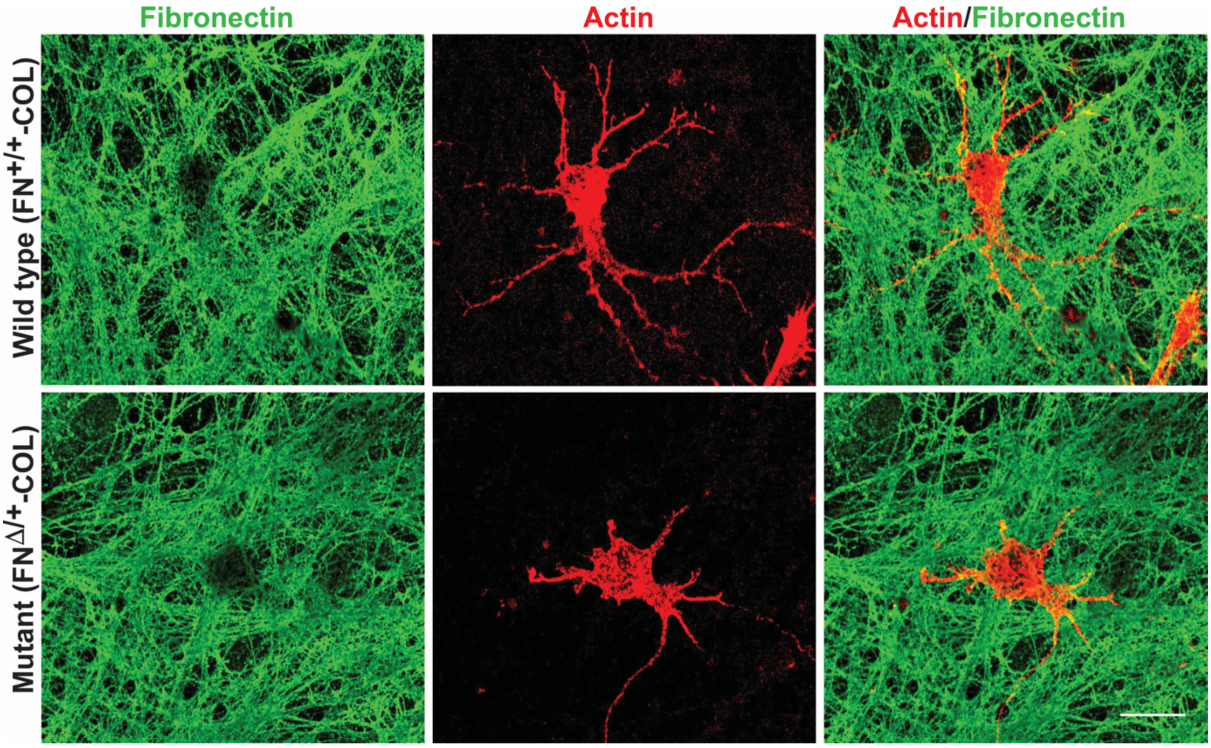

Supplementary Figure 3: Representative confocal images of dendrites extended on wild type and mutant matrices at 72 h. ECM was stained with R184 rabbit anti-fibronectin antiserum followed by goat anti-rabbit IgG (green) and cells were stained with fluorescent phalloidin (red). Regions of overlap between dendrites and fibrils are yellow. Scale bar = 10 µm.

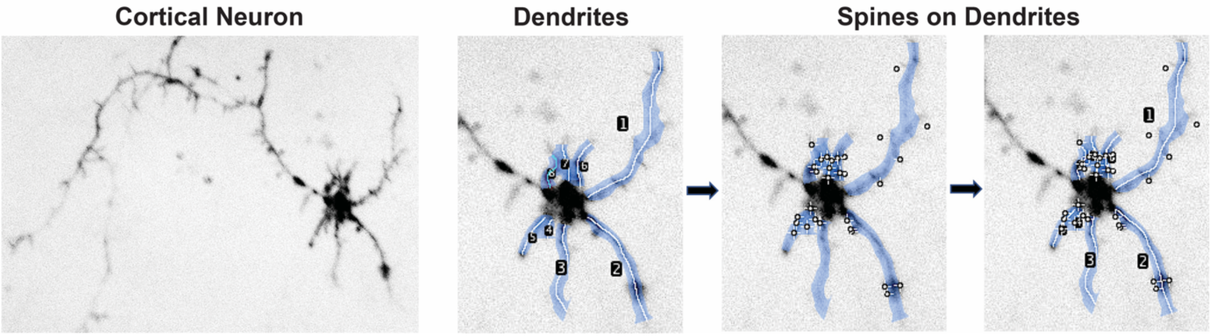

Supplementary Figure 4: Analysis of dendrite outgrowth, the number of dendrites/neuron, dendritic spines, the spine count/neuron on wild type ($FN^{+/+}$-COL) and mutant ($FN^{\Delta/+}$-COL) matrices were measured using "dendritic spine counter" by image J.

Supplementary Table 1: Axon and dendrite lengths

Matrices	No. of Neurons[a]	No. of Axons[b]	Avg. Axon Length (µm)[d]	No. of Neurons[a]	No. of Dendrites[c]	Avg. Dendrite Length (µm)[d]	Total Dendrite Length/Neuron[d]
**$FN^{+/+}$-COL**	44	44	190 ± 17	31	205	11.3 ± 0.7	315.4 ± 10.4
**$FN^{\Delta/+}$-COL**	63	63	130 ± 04	46	172	7.4 ± 0.4	112.4 ± 1.8

[a] Total number of neurons measured separately for axons and dendrites at 72 h of culture on matrices as indicated.
[b] Total number of axons measured.
[c] Total number of dendrites measured.
[d] Average of two independent experiments ± SEM and P values are mentioned in Figure 5.

Supplementary Table 2: Quantification of dendrite and dendritic spine numbers and density

Matrices	No. of Neurons[a]	No. of Dendrites[b]	No. of Spines[c]	Avg. No. of Dendrites/ Neuron[d]	Avg. No. of Spines/Neuron[d]	Avg. Dendrite Width (µm)[d]	Avg. Spine Density (Spines/Dendrite Length (µm))[d]
**$FN^{+/+}$-COL**	31	205	985	7.08 ± 0.05	36.13 ± 4.50	2.95 ± 0.09	0.41 ± 0.06
**$FN^{\Delta/+}$-COL**	46	172	259	3.70 ± 0.30	5.68 ± 0.78	2.82 ± 0.03	0.20 ± 0.03

[a] Total number of neurons measured at 72 h of culture on matrices as indicated.
[b] Total number of dendrites measured at 72 h of culture on matrices as indicated.
[c] Total number of spines measured.
[d] Average of two independent experiments ± SEM and P values are mentioned in Figure 6.

Supplementary Table 3:

List of ECM proteins detected by mass spectrometry

			Total Spectrum Counts			
			Exp.1		Exp.2	
**Gene Name**	**Protein Name**	**MW (kDa)**	**FN^{+}/$^{+}$-COL**	**FN$^{\Delta}$/$^{+}$-COL**	**FN^{+}/$^{+}$-COL**	**FN$^{\Delta}$/$^{+}$-COL**
Fn1	Fibronectin	273 kDa	651	674	556	554
HSPG2	HSPG (Perlecan)	470 kDa	104	109	100	89
Col1a1	Type I-Collagen alpha 1	138 kDa	61	94	39	27
Col6a1	Type VI -Collagen alpha 1	108 kDa	53	48	38	41
Col1a2	Type I-Collagen alpha 2	130 kDa	48	65	48	41
Col6a2	Type VI -Collagen alpha 2	110 kDa	42	30	28	25
Tnc	Tenascin	222 kDa	29	14	14	5
BGN	CSPG (Biglycan)	42 kDa	26	22	26	27
Emilin 1	Emilin	108 kDa	25	21	8	3
Col12a1	Collagen alpha-1(XII)	340 kDa	16	57	44	65
Fbln 2	Fibulin-2	132 kDa	14	16	2	2
Col5a1	Type V -Collagen alpha 1	184 kDa	13	13	5	7
Col5a2	Type V -Collagen alpha 2	145 kDa	9	9	7	3
Anxa 5	Annexin 5	36 kDa	6	13	8	10
Thbs1	Thrombospondin-1	130 kDa	5	4	4	2
